# *Antrodia cinnamomea* Extraction Waste Supplementation Promotes Thermal Stress Tolerance and Tissue Regeneration Ability of Zebrafish

**DOI:** 10.3390/molecules25184213

**Published:** 2020-09-14

**Authors:** Chi-Chang Chang, Yung-Chuan Lu, Chih-Chun Wang, Tsui-Ling Ko, Jung-Ren Chen, Wei Wang, Ya-Ling Chen, Yu-Wen Wang, Tzu-Hsien Chang, Hsia-Fen Hsu, Jer-Yiing Houng

**Affiliations:** 1 School of Medicine for International Students, College of Medicine, I-Shou University, Kaohsiung 82445, Taiwan; ed101779@edah.org.tw (C.-C.C.); gregory.yclu@msa.hinet.net (Y.-C.L.); ccw5969@yahoo.com.tw (C.-C.W.); kate819b@isu.edu.tw (T.-L.K.); 2 Department of Obstetrics & Gynecology, E-Da Hospital, Kaohsiung 82445, Taiwan; igiolal2011@gmail.com; 3 Division of Endocrinology and Metabolism, Department of Internal Medicine, E-Da Hospital, Kaohsiung 82445, Taiwan; 4 Department of Otolaryngology, E-Da Hospital, Kaohsiung 82445, Taiwan; 5 Department of Biological Science and Technology, College of Medicine, I-Shou University, Kaohsiung 82445, Taiwan; jrchen@isu.edu.tw (J.-R.C.); a951753367@gmail.com (W.W.); 6 Department of Nutrition, College of Medicine, I-Shou University, Kaohsiung 82445, Taiwan; sly4128@gmail.com (Y.-W.W.); pt153575@gmail.com (H.-F.H.); 7 Department of Chemical Engineering, I-Shou University, Kaohsiung 82445, Taiwan; andy3560133@gmail.com

**Keywords:** *Antrodia cinnamomea*, zebrafish, thermal stress tolerance, tissue regeneration ability, anti-inflammation

## Abstract

*Antrodia cinnamomea* (AC) has been shown to have anti-inflammatory, anti-tumor, and immunomodulation activities. It is estimated that hundreds of metric tons of AC extraction waste (ACEW) are produced per year in Taiwan. This study aims to assess the feasibility of applying ACEW as feed supplement in the aquaculture industry. ACEW significantly inhibited the growth of microorganisms in the water tank, by around 39.4% reduction on the fifth day with feed supplemented of 10% ACEW. The feed conversion efficiency of zebrafish with 10% ACEW supplementation for 30 days was 1.22-fold compared to that of the control. ACEW dramatically improved the tolerances of zebrafish under the heat and cold stresses. When at water temperature extremes of 38 °C or 11 °C, compared to the 100% mortality rate in the control group, the 10% ACEW diet group still had 91.7% and 83.3% survival rates, respectively. In a caudal fin amputation test, the fin recovery of zebrafish was increased from 68.4% to 93% with 10% ACEW diet after 3-week regeneration. ACEW effectively down-regulated the gene expression of TNF-α, IL-1β, IL-6, and IL-10, and up-regulated the gene expression of IL-4/13A. Additionally, the supplement of ACEW in the feed can maintain and prevent the fish’s body weight from dropping too much under enteritis. Taken together, ACEW has beneficial potential in aquaculture.

## 1. Introduction

Due to health and environmental concerns over the use of chemical drugs, during the past decades, increasing attention has been brought to the application of Chinese herbal medicines. Chinese herbal medicines are generally recognized as being natural and safe with low pollution, no residue, no phytotoxicity, no drug resistance, limited side effects, and have had a long history in the prevention and control of aquaculture diseases [1]. Therefore, the use of Chinese herbal medicines in aquaculture has gained increasing importance, and a variety of literature demonstrates that Chinese herbal medicines have enhanced fish health in many aspects, including growth promotion, increase of disease resistance, and promotion of specific and non-specific immunity [1,2,3].

*Antrodia cinnamomea* (AC), also named *Antrodia camphorata*, *Ganoderma camphoratum*, or *Taiwanofungus camphorata* previously, is a unique and precious endemic Taiwanese medicinal mushroom, known in Chinese as “Niuchangchih”. AC has been used as a traditional medicine for diverse health conditions such as detoxification, liver protection and anti-cancer. Many studies have shown that the biological functions of AC extract include anti-inflammation, anti-tumor, anti-virus, anti-allergy, anti-hypertension, anti-microorganism, immunomodulation, inhibition of platelet aggregation, lowering blood sugar and cholesterol, hepatoprotection, anti-fatigue, and so on [4,5,6]. In terms of human clinical trials, AC has been shown to possess the potential to reduce total cholesterol in healthy adults and has considerable safety. After taking AC for three months, there was no significant change in liver and renal function indices, blood pressure, triglyceride, and fasting blood glucose levels [7]. AC has been used in the complementary treatment of cancers, with some clinical test results reported, including colorectal cancer [8], adenocarcinomas [9], and lung cancer [6,10].

The ingredients of AC encompass triterpenoids, polysaccharides, superoxide dismutase (SOD), adenosine, succinic and maleic acid derivatives, proteins (including immune protein), vitamins, nucleic acid, lectin, ergosterol, and lignin [5,11]. Among them, triterpenoids and polysaccharides are considered as the main sources of AC’s health-care functions, and thus they have become important indicators on quality control of AC.

During the application process of AC, the extraction is usually carried out first, and then the extracted solution or the dried or paste extract is used to prepare the final products. Therefore, many extraction wastes were produced. Due to the booming of this industry recently in Taiwan, the amount of extraction waste has increased dramatically. It is estimated that there should have hundreds of metric tons produced per year and has hence caused environmental concern.

Currently, the zebrafish (*Danio rerio*) has become the second most widely used model animal in human drug development research [12,13]. Zebrafish has also been applied to aquaculture research to investigate fish growth patterns and for scientific purposes, such as disease biology [14,15]. Due to its small size, short generation time, high fertility and, well annotation of its genome, the zebrafish has received increasing attention.

With a goal of finding a sustainable solution for aquaculture development that balances economy and environment, we undertook this study to assess the feasibility of applying the extraction waste of AC (ACEW) to the aquaculture industry. Since a small number of active ingredients are generally left in the raw material residues after extraction, ACEW should still have similar bioactivity as AC. We evaluated the effects of ACEW dietary supplementation on the feed conversion efficiency, heat resistance, cold tolerance, fertility, fin regeneration, and immunity of zebrafish. Each experiment in this study was conducted separately and independently.

## 2. Results

### 2.1. Total Contents of Triterpenoid and Polysaccharide of ACEW

The ACEW used in this study was the residual waste from the extraction of AC by 95% ethanol. In order to analyze the residual composition of ACEW, the powder was further extracted by methanol under sonication. This is a general method for the purpose of phytochemical analysis to evaluate the composition values and for the characterization of vegetal products. The total contents of triterpenoid and polysaccharide of ACEW were analyzed. Compared to the AC ethanol extract, its total contents of triterpenoid and polysaccharide were 309.3 mg/g and 204.6 mg/g, respectively, the ACEW had a much lower amount of triterpenoid (2.91 mg/g) and polysaccharide (42.4 mg/g).

In addition, the proximate composition of ACEW was analyzed as follows: crude protein of 55.0 mg/g, crude lipid of 7.0 mg/g, and carbohydrate of 859 mg/g.

### 2.2. Effect on Water Quality

The water source of the zebrafish aquaculture system is municipal tap water, and the pH value of the tap water after aeration is 8.11 ± 0.02. The initial pH value of the zebrafish aquaculture system for the vehicle, 0.2%, 1%, and 10% ACEW groups were 8.07 ± 0.08, 8.01 ± 0.02, 8.02 ± 0.01, 8.02 ± 0.02, respectively. Different diet groups were fed continuously for 22 days, and one fifth of the water was changed daily after feeding. The pH value was monitored daily. Experimental results show that there was no significant difference in pH between groups during this period (data not shown).

**Figure 1:**
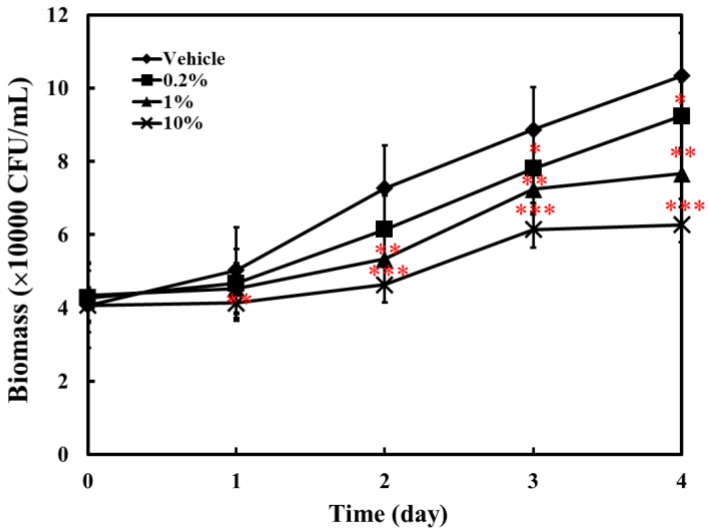
Total bacterial plate count of tank water during 4-day cultivation. Thirty fish were used in each group and placed in the same aquarium with one fifth of tank water changed every day during the feeding experiment period. Significant difference compared with the vehicle group was denoted as * *p* < 0.05, ** *p* < 0.01, and *** *p* < 0.001.

Previous studies have shown that AC extracts have antibacterial and antiviral properties [16,17,18]. Therefore, the effect of ACEW diet supplementation on the inhibition of microbial growth in water was examined. Figure 1 reveals that even with a daily change of one fifth of the tank water, the number of microorganisms in the water increased with the prolongation of the culture time. Notably, the growth of microorganisms in the tank water would be partially inhibited after ACEW was supplemented. The more the ACEW amount was added, the more obvious of this inhibitory effect.

### 2.3. Effect on Growth Performance and Reproduction

**Figure 2:**
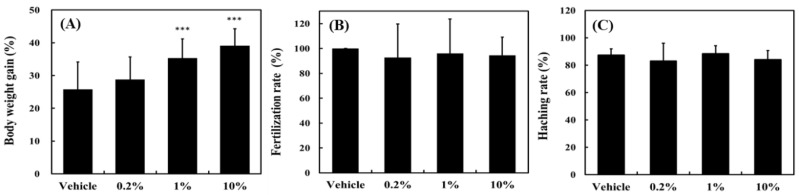
Effect of *Antrodia cinnamomea* extraction waste (ACEW) supplementation on (**A**) weight gain, (**B**) fertilization rate, and (**C**) hatching rate of zebrafish during 30-day cultivation. For the body weight gain study, thirty adult fish were used in each group and put in the same aquarium. For the reproduction study, three males were paired with three female fish for each group in the mating tank one night before spawning event. Significant difference compared with the vehicle group was denoted as *** *p* < 0.001.

Figure 2 shows the effect on zebrafish growth and reproduction after 30 days of ACEW supplementation. Figure 2A shows that supplementation of ACEW promoted the weight gain of zebrafish. Within the range of supplementary levels examined, the higher the supplementation amount was given, the more weight zebrafish gained. However, as indicated in Figure 2B,C, the effect of ACEW supplementation on zebrafish fertilization and hatching rates was insignificant.

A more detailed analysis was conducted with several growth performance indicators as shown in Table 1. Compared with the vehicle group, the growth performance indicators, including body weight gain, specific growth rate, feed conversion efficiency, and daily growth rate, were significantly improved with 1% ACEW supplement dose (*p* < 0.001) or more.

### 2.4. Effect on Tolerance to Rapid Temperature Changes

**Figure 3:**
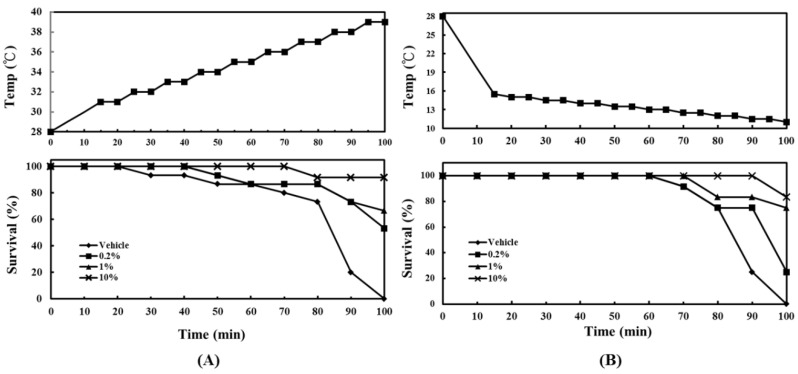
Effect of ACEW supplementation on survival rate of zebrafish under rapid temperature changes. Each group of 12 adult zebrafish was fed with a specified diet for 30 days before the experiment, and then underwent the rapid temperature changes as: (**A**) the heating challenge experiment; (**B**) the cooling challenge experiment. The time course of temperature change is described in Section 4.6.

This experiment used heating or cooling to simulate the extreme environmental temperature changes under heat waves or cold damage, and to examine the effect of ACEW supplement on survival rate of zebrafish. In the heating challenge experiment (Figure 3A), the temperature was raised from 28 °C to 39 °C in 100 min; while in the cooling challenge experiment (Figure 3B), the temperature was reduced from 28 °C to 11.5 °C in 100 min. The experiments were carried out after 30 days of continuous feeding of different diets. As shown in Figure 3A, all fish survived below 31 °C. When the temperature reached 32 °C, fish of the vehicle group began to die. The fish supplemented with 0.2% and 1% ACEW began to die at 33 °C, while the fish supplemented with 10% ACEW began to die at 37 °C, and even when the temperature rose to 39 °C the mortality in this group did not increase. When the temperature reached 39 °C, compared to vehicle group (survival rate = 0%), the survival rates of 0.2%, 1%, and 10% ACEW groups were 58.3%, 66.7%, and 91.7%, respectively.

In the cooling challenge experiment (Figure 3B), all fish survived when the water temperature was decreased from 28 °C to 14 °C. When the temperature continued to drop, only 10% ACEW group could keep all fish alive, while the fish of other groups started to die. When the temperature finally dropped to 11.5 °C, all fish in the vehicle group were dead, while the survival rates of 0.2%, 1%, and 10% ACEW groups were 25.0%, 75.0%, and 83.3%, respectively.

Accordingly, the dietary supplement of ACEW would significantly increase fish’s tolerance to the extreme fluctuations in ambient temperature.

### 2.5. Effect on Caudal Fin Regeneration Ability

**Figure 4:**
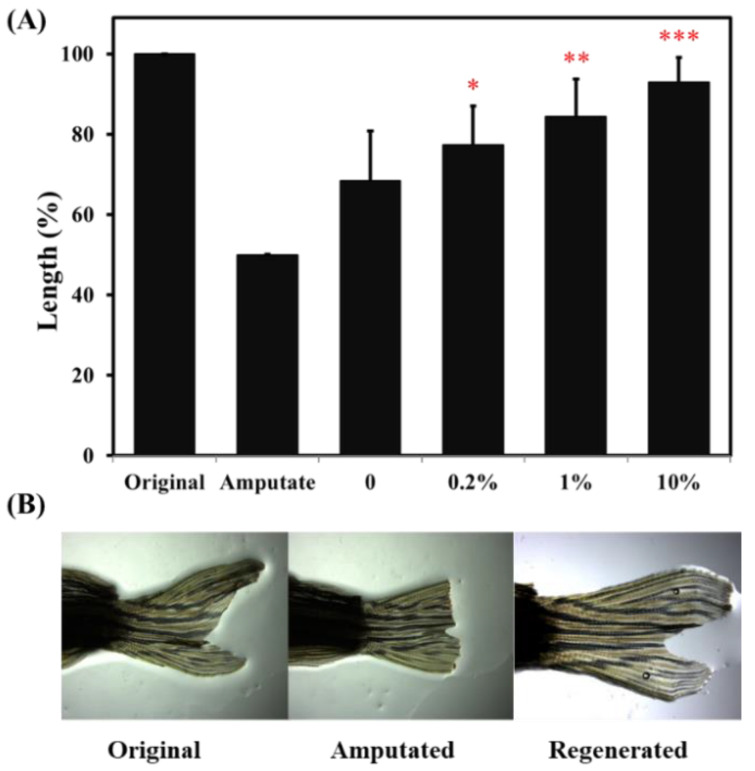
Effect of ACEW supplementation on caudal fin regeneration ability. (**A**) Length changes of caudal fins; (**B**) Typical photographs of the original, amputated and regenerated fins. Each group of 20 adult zebrafish was fed with specified diet for 30 days before the experiment, and then half-length of the caudal fin was amputated. After 21-day recovery, the length of caudal fin was measured. Significant difference compared with the vehicle (group 0) was denoted as * *p* < 0.05, ** *p* < 0.01, and *** *p* < 0.001.

The zebrafish have the fascinating ability to regenerate lost or damaged tissues such as caudal fin. This study compared the regenerative ability of fish by caudal fin amputation and observed their recovery. In this experiment, zebrafish were fed with 0.2%, 1% and 10% ACEW for one month, respectively, and then half of the length of caudal fins were cut off. After three weeks of recovery under normal culture conditions, the caudal fin length was measured to evaluate the fish’s regeneration ability. Figure 4 shows that the supplement of ACEW dramatically increased zebrafish’s regeneration ability dose-dependently. The caudal fin length could be recovered to 68.4 ± 12.3%, 77.3 ± 9.7% (*p* < 0.05), 84.4 ± 9.2% (*p* < 0.01) and 93 ± 6.1% (*p* < 0.001) of the original length for the vehicle, 0.2%, 1% and 10% ACEW groups, respectively.

### 2.6. Anti-Inflammatory Effect

AC has good anti-inflammatory activity [4,5,6]. This experiment explored the anti-inflammatory effect of ACEW on zebrafish by oxazolone-induced intestinal inflammation model [22,23]. Both the prevention mode and the therapeutic mode of ACEW were investigated.

**Figure 5:**
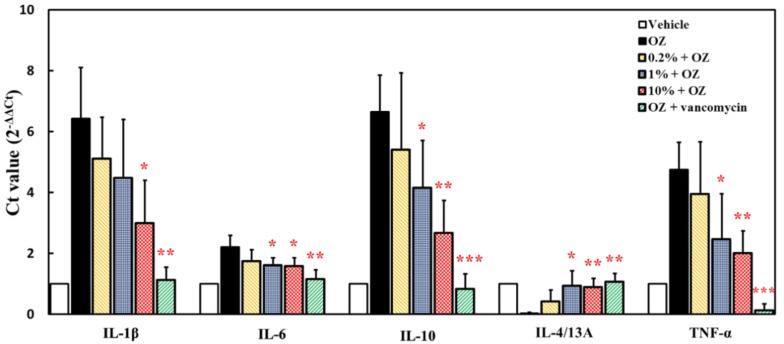
Effect of ACEW supplementation on gene expression of inflammation-associated cytokines in zebrafish under the intestinal stimulation by oxazolone in prevention mode experiment. Each group of 10 adult zebrafish was fed with specified diet for 30 days before the experiment, and then 0.2% oxazolone were intrarectally instilled to induce acute enteritis. Significant difference of gene expression between the ACEW supplemented diet group and the control diet group (group OZ without ACEW addition) was denoted as * *p* < 0.05, ** *p* < 0.01, and *** *p* < 0.001.

In the prevention mode experiment, zebrafish were fed with 0.2%, 1% and 10% ACEW for 30 days, respectively, and then 0.2% oxazolone were intrarectally instilled to induce acute enteritis. As shown in Figure 5, when zebrafish was stimulated with 0.2% oxazolone (group OZ), the gene expression of TNF-α, IL-1β, IL-6, and IL-10 were greatly increased. For the groups with ACEW supplement, the gene expressions of these cytokines were suppressed in a dose-dependent manner. Although the suppressive effects are less than that of the positive control (vancomycin), the ACEW still has the application potential for daily health care on zebrafish. On the other hand, for the gene expression of IL-4/13A, after 0.2% oxazolone induction, its expression level dropped significantly. If ACEW was added to the feed, it could restore the expression level of IL-4/13A to normal value, and this promotion effect was similar to that of the positive control group.

**Figure 6:**
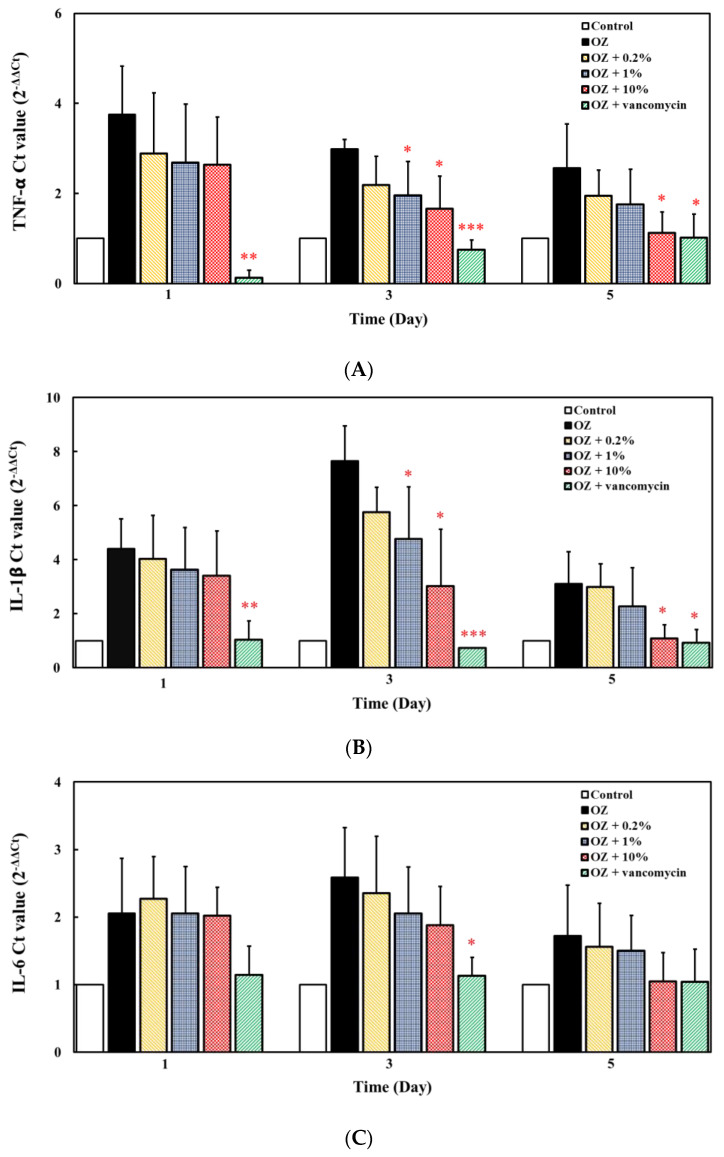

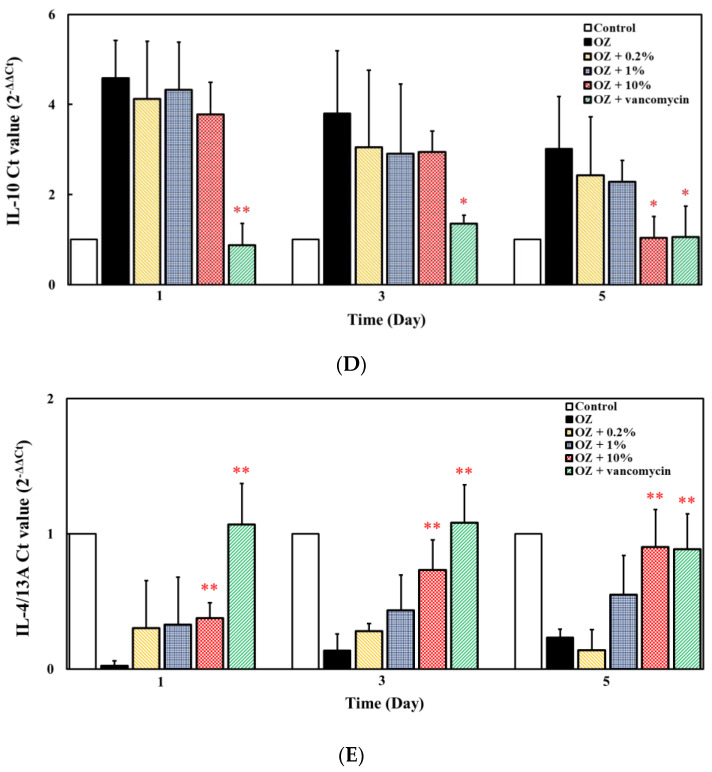
Effect of ACEW supplementation on gene expression of inflammation-associated cytokines in zebrafish under the intestinal stimulation by oxazolone in therapeutic mode experiment. To each group of 10 adult zebrafish, 0.2% oxazolone were intrarectally instilled to induce acute enteritis. After 5-h induction, fish were fed with specified diet twice a day. On day-1, -3, and -5, gene expression of (**A**) TNF-α, (**B**) IL-1β, (**C**) IL-6, (**D**) IL-10, and (**E**) IL-4/13A was analyzed by qRT-PCR. Significant difference of gene expression between the ACEW supplemented diet and the control diet group (group OZ without ACEW addition) was denoted as * *p* < 0.05, ** *p* < 0.01, and *** *p* < 0.001.

In the therapeutic mode experiment, zebrafish were firstly instilled by 0.2% oxazolone to induce acute enteritis, and then different doses of ACEW were fed to evaluate its potential therapeutic action. As shown in Figure 6, positive control vancomycin had significant anti-inflammatory effect after the first day of treatment. Compared to positive control, ACEW had less therapeutic effect unless the ACEW dosage was increased to 10% with the treatment time longer than 3 days.

**Figure 7:**
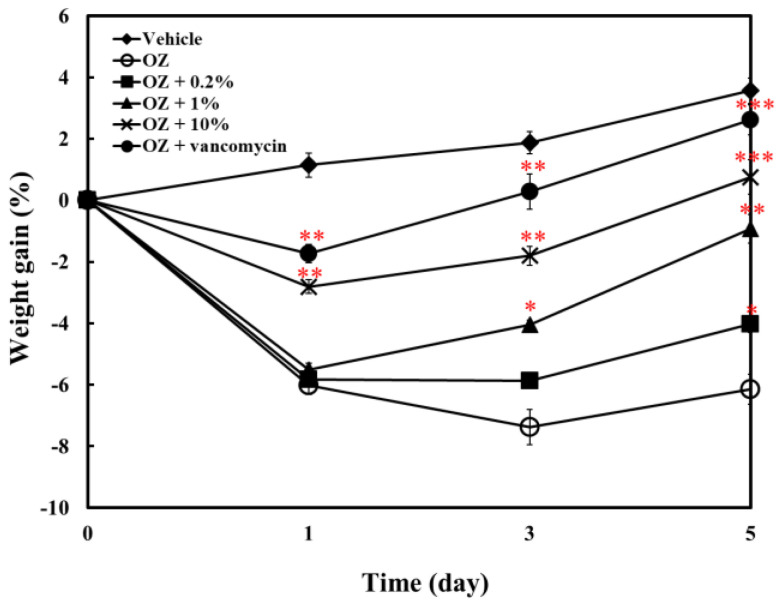
Effect of ACEW supplement on weight gain of zebrafish in therapeutic mode experiment of oxazolone-induced acute enteritis. Zebrafish were fed with different experimental diets after induction, and were weighted at day 1, 3, and 5. Significant difference of gene expression between the ACEW supplemented diet and the control diet group (group OZ without ACEW addition) was denoted as * *p* < 0.05, ** *p* < 0.01, and *** *p* < 0.001.

In addition, as shown in Figure 7, the stimulation of acute enteritis by oxazolone would cause significant decrease in fish’s body weight. The supplement of ACEW in the feed could reduce the symptoms of enteritis, so that the body weight did not drop too much. The average weight of the 10% ACEW group had returned to their initial weight value on the fifth day of treatment.

## 3. Discussion

In 2018, Taiwan’s aquaculture fish production accounted for around 26% of Taiwan’s total fish supply [24]. In order to prevent the reduction of the aquaculture fish production, the prevention and management of fish diseases has become a forefront priority. In recent years, traditionally used medicinal chemicals have caused many negative impacts on the environment and human health. For example, chemical drugs may cause the generation of drug-resistant bacteria strains and residual accumulation in fish tissue. Therefore, increasing attention has been paid to searching alternative natural agents that are environmentally friendly and harmless to the human body. Among them, the use of natural edible plant products for disease control in aquaculture has become an important development direction. Many studies have reported that active molecules such as alkaloids, terpenoids, saponins, and flavonoids in plant products can increase feed intake to promote weight gain, improving feed efficiency, enhancing disease resistance, stimulating immunity and exhibiting antibacterial and antiparasitic properties [1,2,3].

In Taiwan, AC has been widely used in health care [6,11]. However, as it is too expensive to be used in aquaculture, limited studies have investigated the effects of AC on fish health enhancement. Hundreds of tons of AC-extracted residues are generated every year in Taiwan, and hence causes environmental concerns. Given the current circumstances, this study uses the zebrafish model to evaluate the application potential of ACEW as a supplement in aquaculture feeds.

This study shows that the growth of microorganisms in the tank water was inhibited by ACEW feeding in a dose-dependent pattern (Figure 1). AC has the suppressive effect on the growth of microorganisms [16,25]. That may be the reason why the total number of microorganisms in the tank water decreased due to the supplementation of ACEW in feed.

ACEW can significantly increase body weight gain, specific growth rate, feed conversion efficiency, and daily growth rate (Figure 2 and Table 1). The enhancement of body weight and feed conversion efficiency of fish by Chinese medicinal herbs can be attributed to their curative characteristics, including improving the palatability of feed, having unique smell and taste, stimulating the secretion of digestive juices and intestinal motility, promoting intestinal function and nutritional metabolism, increasing intestinal villous length and muscle layer thickness of digestive tract [1,3]. In addition, these enhancements may also be derived from the effects of ACEW’s bioactive ingredients that can promote metabolism, enhancing the synthesis of protein, or activating digestive enzymes [1,3]. The precise mechanism for ACEW to increase fish weight gain still needs further investigation.

Water temperature is a major environmental factor influencing the growth or survival of fish. Fish are frequently affected by a dramatic change in ambient temperature, and thus extreme temperature changes, such as those related to wind, cold, or heat waves, often cause significant economic losses in aquaculture. More specifically, temperature influences the survival and developmental processes of aquatic invertebrates [26,27], as well as their endocrine system [28], immune system [29], oxidative metabolism [30], and physiological and reproductive mechanisms in fish [31,32]. In this study, ACEW supplementation was demonstrated to increase fish’s tolerance to severe changes in ambient temperature (Figure 3). Accordingly, ACEW should be able to improve the overall health of fish.

The zebrafish fin provides a valuable model to assess the organ regeneration ability of fish [33,34]. Regeneration is the process by which damaged or lost structures are perfectly or near-perfectly replaced. After cutting the fin, the wound undergoes rapid re-epithelialization within a few hours, and the fin stump starts the regeneration process immediately after the wound healing. In the present study, after three-week recovery period in post-amputation of caudal fin, feeding ACEW for one month has a great improvement in regeneration ability of fish (Figure 4).

Previous studies have reported that AC extract possesses significant anti-inflammatory activity. These results indicate that AC extract reduced lipopolysaccharide (LPS) induction of inflammatory response by decreasing the expression of inflammation-related genes, including inducible nitric oxide synthase (iNOS), cyclooxygenase-2 (COX-2), IL-6, TNF-α, and NF-κB [35,36,37]. Induction of fish intestinal inflammation is a commonly used research protocol to explore the anti-inflammatory effects of drugs on fish. The stimulation of intestinal inflammation by oxazolone in zebrafish has been widely applied to study the enteritis, immunity and prevention of bacterial infections [22,23,38]. Whether in prevention mode or therapeutic mode, ACEW can effectively reduce the gene expression of TNF-α, IL-1β, IL-6 and IL-10, and increase the gene expression of IL-4/13A, in dose-dependent manner (Figure 5 and Figure 6). These experimental results are similar to the study under the treatment of colistin sulphate [39]. Although the anti-inflammatory effect of ACEW is lower than that of the positive control vancomycin, it does not mean the anti-inflammatory activity of AC extract is not good, as this study aims to explore the feeding effect of its extraction waste. In fact, the concentration of anti-inflammatory components in ACEW has been minimal, but it can still show some inhibitory effect on enteritis of zebrafish. In this regard, ACEW still has anti-inflammatory value as a medical tool to fish. Additionally, the supplement of ACEW in the feed can maintain and prevent the fish’s body weight from dropping too much due to enteritis (Figure 7).

AC is a traditional edible mushroom. It must be finely ground into a powder before extraction, so the ACEW is suitable for fish consumption. In this study, all fish in the experiment survived except for the death of the fish in the thermal stress tolerance experiment. Thus, feeding ACEW is safe for fish.

## 4. Materials and Methods

### 4.1. Preparation of ACEW and ACEW Diets

The fruiting body of AC was obtained from Elohim Biotechnology Development Co. (Kaohsiung City, Taiwan). First, after cutting, the fruiting body was extracted three times with 95% ethanol. After filtration by filter paper, the filtrates were collected, and concentrated with a vacuum evaporator. The AC extract was then obtained by drying it in a freeze-drier (Panchum Scientific Co., Kaohsiung City, Taiwan).

Next, the solid residue was dried in an oven under vacuum to completely evaporate the ethanol and water. The produced solid was ground to a size of approximately 1 mm in diameter. This ACEW powder was then blended with a commercially available fish feed (Talkong Inc., New Taipei City, Taiwan), according to the ratio of 1:499 (0.2% ACEW diet), 1:99 (1% ACEW diet), 1:9 (10% ACEW diet) used in this study. All diets were air-dried and stored in airtight containers at −20 °C until used.

### 4.2. Analysis of Total Triterpenoid Content and Total Polysaccharide Content

When analyzing ACEW, the sample was first extracted with seven volumes of methanol in ultrasonic for 20 min. The particles were removed by centrifugation. The total triterpenoid content was determined according to Chang et al. [40] with some modifications. An aliquot of 0.1 mL sample, 0.4 mL of 5% vanillin-glacial acetic acid solution, and 0.8 mL perchloric acid solution was heated at 60 °C for 15 min and then cooled in 4 °C water bath for 2 min. After the addition of 5 mL glacial acetic acid for 10 min, the absorbance of the solution was measured against a blank at 550 nm. A calibration curve was constructed using ursolic acid as a standard. The total triterpenoid content is expressed as milligram ursolic acid equivalents per gram of sample.

The total polysaccharide content was determined according to Pawar and D’Mello [41] with some minor modifications. To an aliquot of 1 mL sample, 1 mL of 5% phenol solution and 5 mL of concentrated sulfuric acid solution were added and mixed thoroughly. The absorbance was measured after 30 min at 488 nm against blank. A calibration curve was constructed using D-glucose as a standard. The total polysaccharide content is expressed as milligram glucose equivalents per gram of samples.

### 4.3. Fish Husbandry

The animal experiments were conducted according to the regulations of local and central government, and were approved by the Institutional Animal Care and Use Committee of I-Shou University (AUP-105-43-06).

The adult captive-bred strain of wild-type zebrafish was purchased from a local pet shop (Kaohsiung, Taiwan) and acclimated to laboratory conditions for at least 2 wk before experiments. The fish was reared at 28 ± 2 °C and under a photoperiod regime of 14/10 h light/dark. Water quality was maintained by circulation with a Fluval U2 aquarium internal filtration system (Rolf C. Hagen Inc., Montreal, QC, Canada). The diet was administered twice daily with a fixed amount of 0.025 g/fish/meal, and one fifth of the water was exchanged daily after feeding.

Different groups of approximately 6-month-old adult fish were randomly allotted separately to each glass aquarium (each with a dimension of 50 cm × 30 cm × 30 cm). The number of fish used in each group of experiments is indicated in each experimental method. All experiments were performed in triplicate independent experiments. Each experiment was conducted separately and independently.

### 4.4. Determination of Water Quality Changes

In this experiment, thirty fish were used in each group and put in the same aquarium. In order to determine whether the ACEW will affect the water quality of the aquariums, 5 mL of water sample, in triplicate, from each aquarium was collected twice daily, 30 min after each feeding. The pH value was measured with a pH meter (PB-10, Sartorius, Göttingen, Germany), and the number of bacteria was determined by total plate count method as follows.

The medium used in total plate count measurement included 0.5% tryptone, 0.25% yeast extract, 0.1% glucose and 2% agar. The sterilized medium was poured onto the petri dish and placed the medium into solid. The diluted water sample was spread into the petri dish in triplicate and incubated at 36 °C for 48 h. The total number of colonies was calculated and expressed as colony-forming units (CFU/mL).

### 4.5. Zebrafish Spawning and Embryo Collection

In this experiment, each group contained three male fish and were paired with three female fish in the mating tank the night before spawning event. Males and females were housed in different chambers separated by a transparent plastic divider, from the start of dark cycle. In the morning, with the beginning of light cycle, the barrier between males and females was removed to initiate spawning. Total spawned eggs and fertilized eggs were counted to calculate the fertilization rate. The hatching rate was calculated as the percentage of hatched eggs after 72 hpf of the total living eggs as follows:

Hatching rate = (hatched embryos/total embryos) × 100%

The fertilization rate is defined as the ratio of fertilized eggs to total spawned eggs as follows:

Fertilization rate = (fertilized eggs/total number of eggs) × 100%

where fertilized eggs = total number of eggs–Infertile eggs.

### 4.6. Temperature Challenge Tests

In order to determine whether the zebrafish increased their tolerance to temperature change after feeding ACEW, two thermal cycle experiments were performed, one for abnormal temperature rise and the other for abnormal temperature drop. Twelve adult zebrafish were placed in a cylindrical glass container with a circulating water jacket. The diameter and the height of the inner cylinder was 20 cm and 80 cm, respectively. The water temperature was controlled by a water circulation system through the jacket with a thermal couple.

In order to simulate the abnormal temperature rise, the temperature of the culture water was increased from 28 °C to 31 °C in 15 min and maintained at this temperature for 5 min. Then, the temperature rose 1 °C per 5 min and kept for 5 min. This continued until the temperature reached 39 °C at 100 min. On the other hand, in the experiment of abnormal temperature drop, the temperature of the water body was decreased from 28 °C to 15.5 °C in 15 min and kept for 5 min. Then, the temperature dropped 0.5 °C per 5 min, and continued until the temperature dropped to 11.5 °C at 100 min. During the experiments, the survival rates of zebrafish were recorded.

### 4.7. Regeneration Ability—Caudal Fin Recovery

Zebrafish were fed with 0%, 0.2%, 1%, and 10% ACEW for one month, respectively. Twenty adult fish were used in each group. Half of the length of caudal fins of all fish were cut off. Cultivated and fed with the same previous diets during the recovery period. After three weeks, the caudal fin length of each fish was measured.

### 4.8. Anti-Inflammatory Effect—Oxazolone-Induced Enteritis

The prevention mode and the therapeutic mode of ACEW supplement were investigated. In the prevention mode experiment, after zebrafish were fed with 0.2%, 1%, and 10% ACEW for one month, respectively, 10 adult fish were anesthetized with 0.05%(*w*/*v*) tricaine (Sigma-Aldrich, St. Louis, MO, USA), and injected 0.2% oxazolone (4-ethoxymethylene-2-phenyl-2-oxazolin-5-one; Sigma-Aldrich, dissolved in 50% ethanol) or 50% ethanol only (vehicle), in a total volume of 0.6 μL/100 mg body weight, which was rectally instilled with a 20 μL Hamilton syringe equipped with a blunt-end needle (Hamilton Co., Reno, NV, USA). Vancomycin (10 μg/mL) was used as the positive control. After 5-h induction of acute enteritis, fish were anesthetized with 0.05%(*w*/*v*) tricaine, the intestinal tissue was dissected, and gene expression of related inflammatory cytokines was analyzed by qRT-PCR.

In the therapeutic mode experiment, 10 adult fish were anesthetized, and 0.2% oxazolone of 0.6 μL/100 mg body weight was rectally instilled. After 5-h induction of acute enteritis, fish were fed with 0.2%, 1%, and 10% ACEW, respectively. On day-1, -3, and -5, gene expression of related cytokines was analyzed by qRT-PCR (Applied Biosystems, Waltham, MA, USA).

### 4.9. Gene Expression Analysis with Quantitative RT-PCR

The zebrafish intestinal tissue was kept at −20 °C until processing. RNA was isolated using the Qiagen RNeasy Kit (Qiagen AG, Venlo, The Netherlands) according to the manufacturer’s protocol. Reverse transcription of mRNA into cDNA was performed using the Magic RT cDNA synthesis kit (Bio-Genesis, Taipei, Taiwan). The obtained cDNA was amplified by IQ2 SYBR Green Fast qPCR System Master Mix LOW ROX kit (Bio-Genesis, Taipei, Taiwan) with ABI 7500 Real-Time PCR System (Applied Biosystems, Foster City, CA, USA). The qPCR reactions started with a holding stage at 50 °C for 2 min, then another holding stage at 95 °C for 10 min. A total of 40 cycles were run (each 95 °C for 15 s, followed by 60 °C for 1 min). The specific primers used for qRT-PCR are listed in Table 2. Amplification of β-actin mRNA was used as an internal control. Each sample was conducted in triplicate. After the PCR program, qRT-PCR data were analyzed by IQ5 Optical System Software (Bio-Rad, Hercules, CA, USA). The baseline was set automatically by the software. The relative expression levels of target genes to the control were analyzed by 2^−^^△△Ct^ relative quantification method [42].

### 4.10. Statistical Analysis

All experiments were performed in triplicate independent experiments and the data obtained were expressed as Mean ± SD. Statistical differences were analyzed by Student’s *t*-test (* *p* < 0.05, ** *p* < 0.01 and *** *p* < 0.001). The experimental data were analyzed using Microsoft Excel software (Office 2019, Microsoft Software Inc., Redmond, WA, USA).

## 5. Conclusions

This study evaluates the feasibility of applying ACEW in aquaculture feeds by using zebrafish as model organism. Experimental results demonstrate that ACEW could significantly inhibit the growth of microorganisms in the cultivation tank. With the supplementation of ACEW, the feed efficiency of zebrafish was increased remarkably, the tolerance to dramatic temperature changes was improved, and the fish’s regeneration ability was enhanced. Moreover, ACEW could reduce fish’s symptoms under inflammatory diseases due to its anti-inflammatory activity. In summary, ACEW has proven potential for application as supplement in aquaculture feeds.

## Figures and Tables

**Table 1:** Growth performance of zebrafish fed diets supplemented with different doses of ACEW ^a^.

Growth Indices	Vehicle	0.2% ACEW	1% ACEW	10% ACEW
Body weight gain (∆W, g) ^b^	0.082 ± 0.029	0.086 ± 0.024	0.099 ± 0.021 ***	0.107 ± 0.018 ***
Specific growth rate (μ, day^−1^) ^c^	0.809 ± 0.095	0.859 ± 0.075 *	1.008 ± 0.082 ***	1.084 ± 0.082 ***
Feed conversion efficiency (FCE, %) ^d^	11.87 ± 1.55	11.67 ± 1.67	13.67 ± 1.72 ***	14.47 ± 1.08 ***
Daily growth rate (DGR, %) ^e^	0.917 ± 0.12	0.981 ± 0.10 *	1.178 ± 0.10 ***	1.282 ± 0.11 ***

^a^ Feed amount: 0.025 g/fish/meal for 30 days (*n* = 30). A significant difference between the vehicle and the ACEW-supplemented diet experiment was indicated as * *p* < 0.05; and *** *p* < 0.001. ^b^ ∆W = W*_f_* − W*_i_*; W*_f_*: final weight, W*_i_*: initial weight. ^c^ μ = (Ln W*_f_* − Ln W*_i_*)/day [19]. ^d^ FCE = ∆W/feed × 100% [19,20]. ^e^ DGR = (∆W/W*_i/_*days) × 100% [21].

**Table 2:** The primers used for detecting cytokine expression in RT-PCR.

Primer	Sequence	Reference
β-actin	5′-CACCATGAAGATCAAGATCA-3′	
	5′-TTTATTCAAGATGGAGCCACCGATCC-3′	
TNF-α	5′-AAGGAGAGTTGCCTTTACCG-3′	[43]
	5′-ATTGCCCTGGGTCTTATGG-3′	
IL-1β	5′-TGGACTTCGCAGCACAAAATG-3′	[44]
	5′-GTTCACTTCACGCTCTTGGATG-3′	
IL-6	5′-TCAACTTCTCCAGCGTGATG-3′	[44]
	5′-TCTTTCCCTCTTTTCCTCCTG-3′	
IL-10	5′-TCACGTCATGAACGAGATCC-3′	[45]
	5′-CCTCTTGCATTTCACCATATCC-3′	
IL4/13A	5′-AGTCACGCTGCTGATGAAGA-3′	[46]
	5′-AACTTGGTCTTGGGCTTTTT-3′	
